# Lung microbiome alterations in NSCLC patients

**DOI:** 10.1038/s41598-021-91195-2

**Published:** 2021-06-03

**Authors:** Leliang Zheng, Ruizheng Sun, Yinghong Zhu, Zheng Li, Xiaoling She, Xingxing Jian, Fenglei Yu, Xueyu Deng, Buqing Sai, Lujuan Wang, Wen Zhou, Minghua Wu, Guiyuan Li, Jingqun Tang, Wei Jia, Juanjuan Xiang

**Affiliations:** 1 Hunan Cancer Hospital, The Affiliated Cancer Hospital of Xiangya School of Medicine, Central South University, Changsha, Hunan China; 2 Cancer Research Institute, School of Basic Medical Science, Central South University, Changsha, Hunan China; 3 Department of Thoracic Surgery, The Second Xiangya Hospital, Central South University, Changsha, 410013 Hunan China; 4 Hong Kong Phenome Research Centre, School of Chinese Medicine, Hong Kong Baptist University, Kowloon Tong, Hong Kong China; 5 Department of Pathology, The Second Xiangya Hospital, Central South University, Changsha, 410013 Hunan China; 6 NHC Key Laboratory of Carcinogenesis and the Key Laboratory of Carcinogenesis and Cancer Invasion of the Chinese Ministry of Education, Xiangya Hospital, Central South University, Changsha, Hunan China; 7 Hunan Key Laboratory of Nonresolving Inflammation and Cancer, Changsha, 410013 Hunan China

**Keywords:** Cancer, Immunology, Microbiology

## Abstract

Lung is colonized by a diverse array of microbes and the lung microbiota is profoundly involved in the development of respiratory diseases. There is little knowledge about the role of lung microbiota dysbiosis in lung cancer. In this study, we performed metagenomic sequencing on bronchoalveolar lavage (BAL) from two different sampling methods in non-small cell lung cancer (NSCLC) patients and non-cancer controls. We found the obvious variation between bronchoscopy samples and lobectomy samples. Oral taxa can be found in both bronchoscopy and lobectomy samples and higher abundance of oral taxa can be found in bronchoscopy samples. Although the NSCLC patients had similar microbial communities with non-cancer controls, rare species such as *Lactobacillus rossiae*, *Bacteroides pyogenes*, *Paenibacillus odorifer*, *Pseudomonas entomophila*, *Magnetospirillum gryphiswaldense,* fungus *Chaetomium globosum *et al*.* showed obvious difference between NSCLC patients and non-cancer controls. Age-, gender-, and smoking-specific species and EGFR expression-related species in NSCLC patients were detected. There results implicated that different lung segments have differential lung microbiome composition. The oral taxa are found in the lobectomy samples suggesting that oral microbiota are the true members of lung microbiota, rather than contamination during bronchoscopy. Lung cancer does not obviously alter the global microbial composition, while rare species are altered more than common species. Certain microbes may be associated with lung cancer progression.

## Introduction

Many regions of the human body, including the gastrointestinal tract, skin, oral cavity, lung, and reproductive tract, are colonized by a diverse community of microorganisms, referred to as the human microbiota^1^. These microbes include bacteria, archaea, fungi, protists, and viruses that affect host physiology, and the compositional alterations of them can disrupt host homeostasis. Healthy lungs that were previously thought to be sterile are now known to harbor a diverse microbiota^2^. Culture-independent methods based on nucleic acid 16S rRNA sequences show that the lower respiratory tract contained many types of bacteria^3^.

Although there is less bacterial biomass in lungs compared to the intestinal tract, the microbiota of the lung displays considerable diversity, and the lung microbiota is similar to that of the duodenum at the phylum level^4,5^. The predominant phyla in healthy lungs are *Bacteroidetes*, particularly *Prevotella* spp. and *Firmicutes*^6^. The phyla of microbiota in the lungs have been shown to shift from *Gammaproteobacteria* and *Firmicutes* towards *Bacteriodetes* in the first 2 weeks of life^4^. The lung is continually exposed to microbes both from environment and the upper airway^7,8^. Healthy lower airways contain bacteria that are distinct from the upper respiratory tract^7^. However, controversy remains on the existence of distinct organisms in the lungs and whether upper respiratory contamination occurs because of passing a bronchoscope through the oral cavity^8,9^.

Lung cancer is the most common cause of cancer-related death worldwide. Patients with lung cancer have an increased risk of microbial infection^10^. It has been increasingly recognized that repeated microbial exposure reshape the lung immune system^11^ and the roles of pathogens in the lung disease have been intensively explored^12^. Inflammation caused by microbial infection may contribute to cancer development and progression^12^. *Staphylococcus* spp.*, Bacillus* spp.*, Haemophilus influenza,* and *Candida albicans* have been identified in lung cancer patients with various degrees of lung inflammation^13^. Possible etiological roles of the compositional balance of lung microbiota in lung cancer are of great interest^14^. The microbial composition of the lower airway in the lung cancer patients is significantly different from that of the non-cancer controls, which is associated with up-regulation of ERK and PI3K signaling pathways^15^. The lower airway dysbiosis occurs more in advanced lung cancer compared to early stage of lung cancer. *Veillonella parvula* was identified as the most abundant taxa, causing decreased survival and increased tumor burden^16^.

In this study, we compared the microbiome of lower respiratory tract from different sampling methods in NSCLC patients without clinical evidence of infection. Because the lung microbiome has a relatively low biomass, the detection of bacterial DNA and RNA and contamination of the BAL samples can be problematic. Therefore, instead of 16S rRNA sequencing based on PCR, we collected bronchoalveolar lavage (BAL) fluid samples through 2 types of sampling methods that represent BAL from different anatomy sites and performed shotgun metagenomic sequencing to identify the specific microbiota in NSCLC patients. These results represent a comprehensive evaluation of lung microbiome in NSCLC to date.

## Results

### The overall profiles of lung microbiome

**Figure 1:**
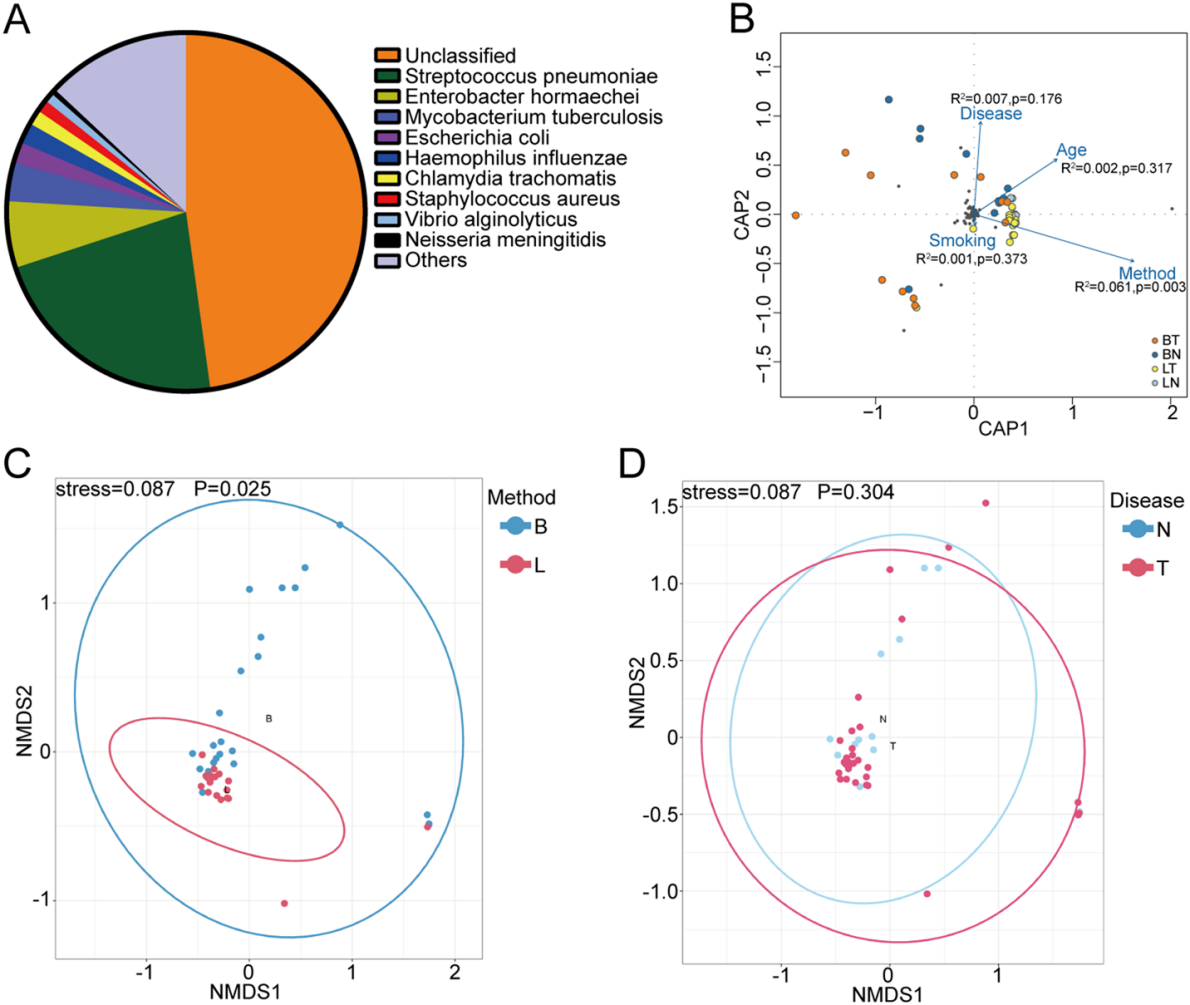
Overall microbiome composition of lungs. (**A**) Taxonomic composition of non-human sequences at species level across 47 samples. (**B**) Distance based redundancy analysis (RDA) with 4 explanatory variables age, sampling methods, disease and smoking history affecting the microbial community composition. Variables were plotted as points. NMDS analysis of morphological variation of 47 samples collected by 2 sampling methods in the NSCLC patients and non-cancer controls were shown in (**C**) and (**D**). Colorful dots indicate different sampling methods or disease cases. Dotted lines display ellipses, which represent the 95% confidence interval. Anosim was performed to test statistically whether there is a significant difference. p < 0.05 was considered to be significant. *B* bronchoscopy samples, *L* lobectomy samples, *N* non-cancer controls, *T* NSCLC patients.

To elucidate the lung microbiome in NSCLC patients, we performed shotgun metagenomic sequencing on 47 samples, 15 of which were from non-cancer controls and 32 of which were from NSCLC patients (Table 1). Analyses were performed on bronchoalveolar lavage (BAL) from patients who underwent bronchoscopy (25/47) or lobectomy (22/47). The lobectomy BAL samples were collected from alveoli through lobes without through the upper airway. We obtained about 15 GB of reads for each sample. The metagenomic sequencing approach resulted in the identification of a species-level sensitivity in all samples (100%) (47/47 samples). Raw sequence data were filtered to eliminate low-quality (contig number < 6000) and human host sequenced reads. The mapping rates varied from 4.35 to 58.4%, indicating that there was some human DNA contamination. There is no significant difference in mapping rates between two sampling methods (data not shown). Reads identified as kingdom were found to be of bacterial, eukaryotic, viral and archaic origin. Taxonomic assignments of operational taxonomic units revealed a total of 1444 bacterial, 270 fungal, 103 viral, 27 archaeon species across all samples. For the following analysis, the taxa with frequency less than 10% of these 47 samples were discarded. Overall, the dominant phyla were *Firmicutes*, *Proteobacteria* and *Actinobacteria* and the dominant genus were *Streptococcus*, *Enterobacter* and *Mycobacterium* (data not shown). Of the species identified, *Streptococcus pneumonia*, *Enterobacter hormaechei* and *Mycobacterium tuberculosis* were the most common (Fig. 1A). We assessed how different variables including age, sampling methods, disease and smoking history contribute to the observed patterns in microbial community using redundancy analysis (RDA). Distance based RDA revealed a higher correlation between microbial community composition (response variables) and explanatory variable sampling method (R2 = 0.061, p = 0.003). However, disease, smoking history and gender did not show obvious correlations with overall microbial composition (Fig. 1B). We used nonmetric multidimensional scaling (NMDS) analysis based on Bray–Curtis distance to compare the community composition between different sampling methods and NSCLC patients or non-cancer controls. NMDS ordination with 95% confidence interval ellipses revealed a better differentiation in the microbial communities between bronchoscopy samples and lobectomy samples than that between NSCLC patients and non-cancer controls (Fig. 1C,D). It revealed separation of bronchoscopy samples from lobectomy samples, while the microbial composition did not differ between NSCLC patients and non-cancer controls.

**Table 1:** Clinical characteristics of patients.

Clinical characteristics of patients	
**Patients (n = 47)**	
NSCLC patients	32 (68.1%)
Non-cancer controls	15 (31.9%)
**Genders (n = 47)**	
Male	26 (55.3%)
Female	21 (44.7%)
**Age (n = 47)**	
≤ 60	27 (57.4%)
> 60	20 (42.6%)
**Sampling methods (n = 47)**	
Bronchoscopy	25 (53.2%)
Lobectomy	22 (46.8%)
**Smoking status (n = 46)**	
Smoker	34 (72.3%)
Non-smoker	12 (26.7%)
**EGFR expression (n = 32)**	
Low (≤ 50%)	6 (18.8%)
High (> 50%)	26 (81.2%)
**TNM stage (n = 32)**	
I–II	23 (71.9%)
III–IV	9 (28.1%)

### The microbial composition varied in different anatomy sites

**Figure 2:**
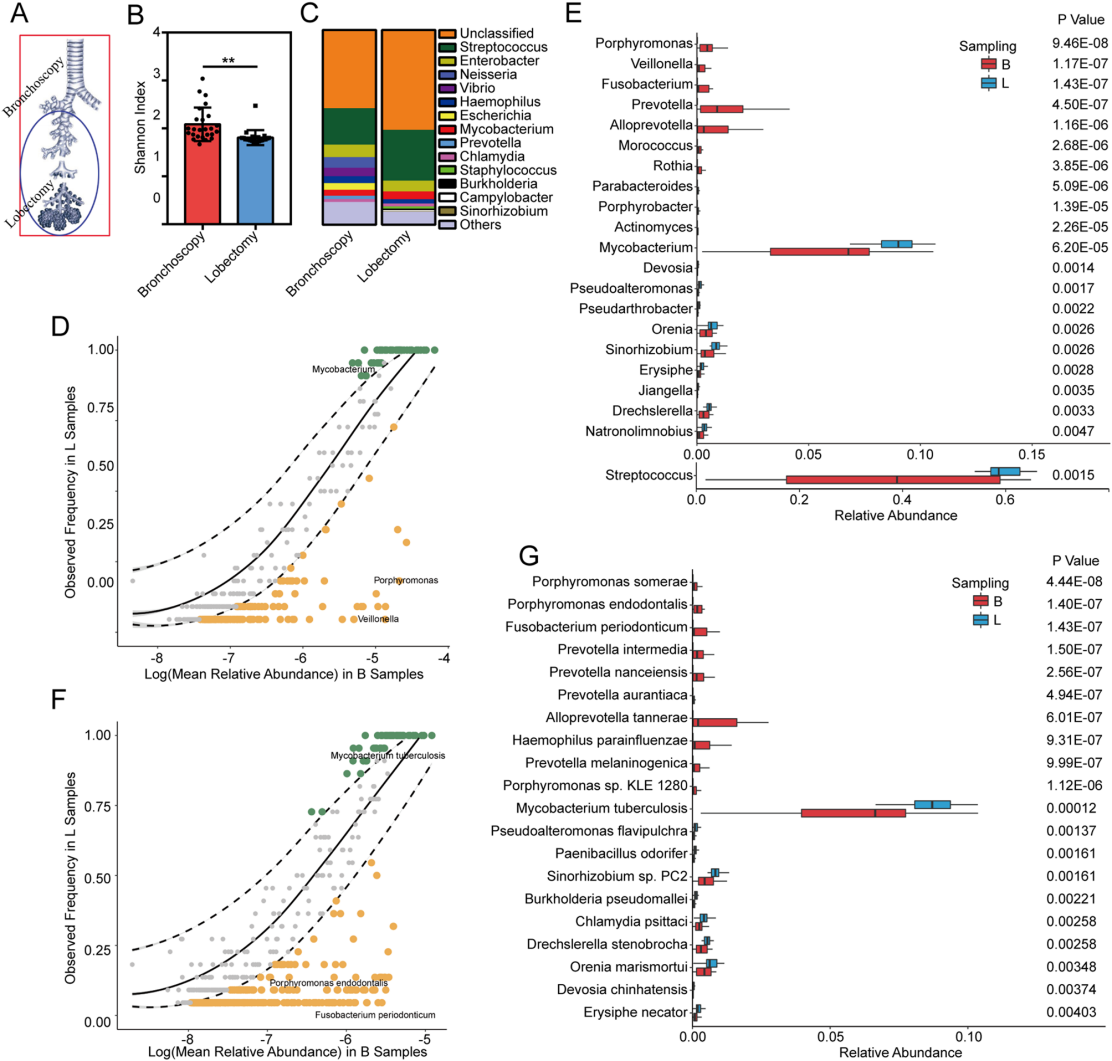
The microbial composition varied in different anatomy sites. (**A**) The schematic diagram of the sampling sites. The squared area represents the extent of bronchoscopy sampling area. The circled area covers the sampling area from lobectomy. (**B**) The Shannon diversity index of bronchoscopy samples and lobectomy samples. Difference between sampling methods was determined by Student t test. ***p < 0.001. (**C**) Taxonomic composition at phylum level in bronchoscopy and lobectomy samples. Neutral model applied to compare the overall lung microbiota composition at genus level (**D**) and at species level (**E**). Dashed lines represent 95% confidence intervals around the model prediction (solid line). The green dots that are beyond the 95% confidence intervals are the OTUs whose observed frequency in lobectomy samples is greater than the model prediction (bronchoscopy samples). The dark golden points that are beyond the 95% confidence intervals are the OTUs whose observed frequency in lobectomy samples is less than the model prediction (bronchoscopy samples). (**F**)Taxonomic composition of lobectomy samples at genus level in NSCLC patients and non-cancer controls. The differential genes between NSCLC patients and non-cancer controls in lobectomy samples were identified by Wilcoxon rank-sum test (p < 0.05). (**G**) Taxonomic composition of lobectomy samples at genus level in NSCLC patients and non-cancer controls. The differential genes between NSCLC patients and non-cancer controls in lobectomy samples were identified by Wilcoxon rank-sum test (p < 0.05). *B* bronchoscopy samples, *L* lobectomy samples.

In order to determine the microbiome variation between different lung anatomy sites, we compared the composition of the microbiota of BAL collected from bronchoscopy and lobectomy. The sampling sites were presented in Fig. 2A. The squared area represents where bronchoscopy reaches. The circled area covers the sampling area from lobectomy. Twenty-two lobectomies and 25 bronchoscopies were performed in these 47 individuals. There was no difference in mapping rates between these two sampling methods (student t test, p > 0.1), indicating that non-related DNA from host exist in both methods. The Shannon diversity index showed that bronchoscopy samples were more diverse than lobectomy samples, suggesting that bronchoscopy samples may contain more microbes from upper airway than lobectomy samples (Fig. 2B). Across 25 bronchoscopy samples, the most common genera were *Streptococcus, Neisseria* and *Enterobacter*. Across all 22 lobectomy samples, the most common genera were *Streptococcus, Enterobacter* and *Mycobacterium* (Fig. 2C). We used neutral model with the bronchoscope samples as a source of OTUs found in the lobectomy samples to evaluate if microbial community in lungs is region specific^17^. Some of the microbiota community observed in bronchoscope samples overlapped with the microbiota community in lobectomy (Fig. 2D,E). However, we can find obvious difference in some species in different sampling methods. The green dots that are beyond the 95% confidence intervals are the OTUs whose observed frequency in lobectomy samples is greater than the model prediction (bronchoscopy samples). The dark golden points that are beyond the 95% confidence intervals are the OTUs whose observed frequency in lobectomy samples is less than the model prediction (bronchoscopy samples). A Wilcoxon t test was performed on the mean relative abundance of these OTUs at genus and species levels. A higher abundance of *Porphyromonas*, *Veillonella*, *Fusobacterium*, *Prevotella *et al*.* were detected in bronchoscopy samples compared to the lobectomy samples (Fig. 2F, supplementary table 1). At species level, *Porphyromonas somerae, Porphyromonas endodontalis*, *Fusobacterium periodonticum*, et al. were increased in bronchoscope samples compared to lobectomy samples (Fig. 2G, supplementary table 2). It suggested that bacteria commonly found in oral cavity such as *Streptococcus, Veillonella* et al. were dispersed from upper airway to lower airway*. Mycobacterium tuberculosis*, *Pseudoalteromonas flavipulchra*, *Streptococcus pneumoniae. PC2* et al. were overpresented in lobectomy samples compared to the bronchoscope samples (Fig. 2G, supplementary table 2). It suggested that these bacteria reach the terminal alveoli after being inhaled into the lung or inhabits in lower part of bronchi. From above, we suggested the regional variation of the lung microbiota and the bronchioles and alveoli may eliminate the microorganisms that aspirated from external environment and oral cavity.

### Microbiome composition of NSCLC patients and non-cancer controls in bronchoscopy samples

**Figure 3:**
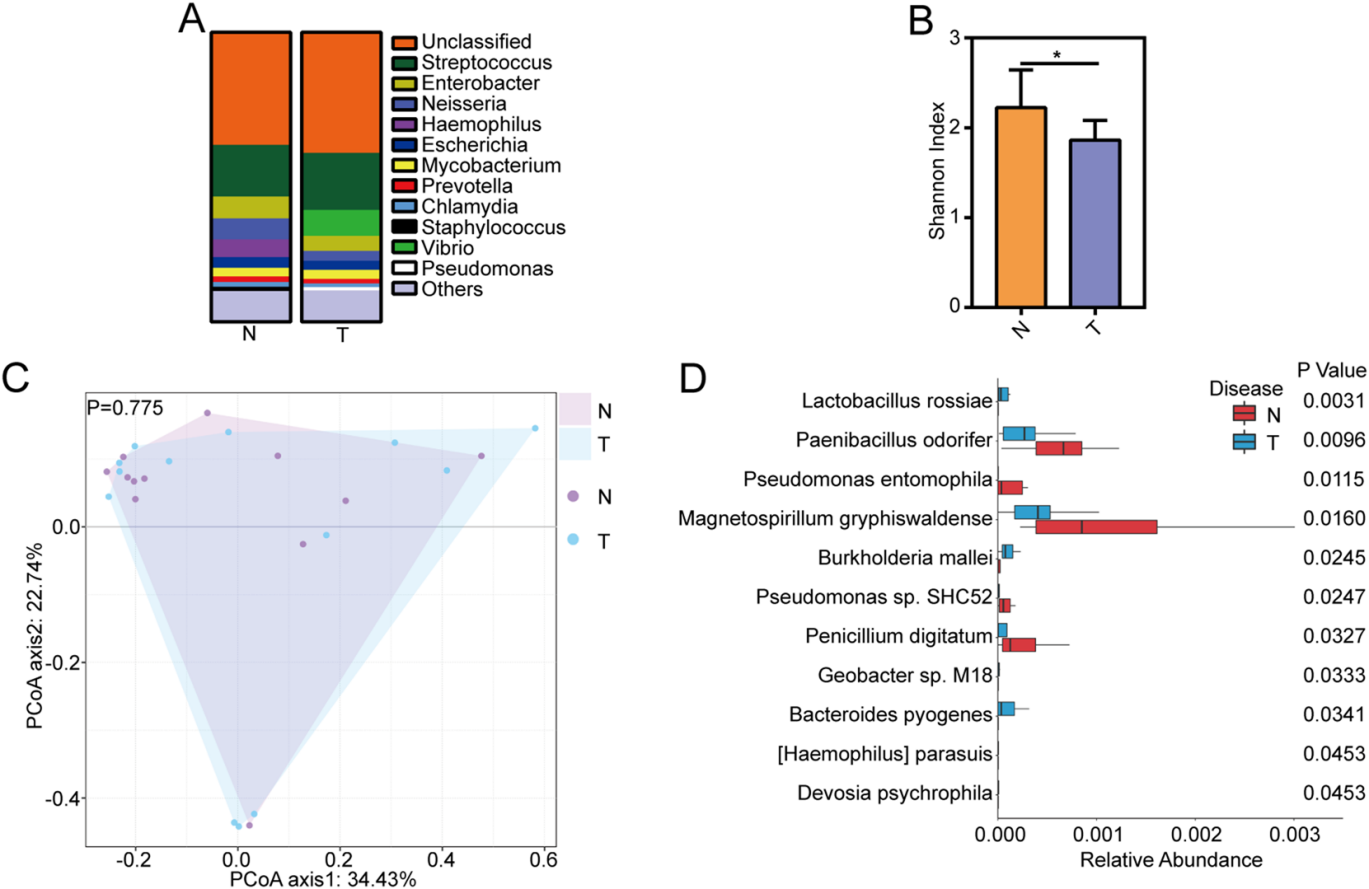
Microbiome composition of NSCLC patients and non-cancer controls in bronchoscopy samples. (**A**) Taxonomic composition of non-human sequences at phylum level in NSCLC patients and non-cancer controls in bronchoscopy samples. (**B**) The Shannon diversity index between NSCLC patients and non-cancer controls in bronchoscopy samples. Difference between sampling methods was determined by Student t test. *p < 0.05. (**C**) PCoA analysis of NSCLC patients and non-cancer controls in bronchoscopy samples. (**D**) The differential species between NSCLC patients and non-cancer controls in bronchoscopy samples were identified by Wilcoxon rank-sum test (p < 0.05). *N* non-cancer controls, *T* NSCLC patients.

As the obvious variation on lung microbiota in different sampling methods, we compared the difference between NSCLC patients and non-cancer controls in different sampling methods. In bronchoscopy samples, across 12 non-cancer controls samples, the most common genera were *Streptococcus, Enterobacter and Neisseria*. Across 13 NSCLC patient samples, the most common genera were *Streptococcus, Vibrio* and *Enterobacter* (Fig. 3A). The Shannon diversity index showed that non-cancer controls have more diverse lung microbiota than NSCLC patients (Fig. 3B). The principal coordinate analysis (PCoA) suggested that NSCLC patients and non-cancer controls had similar microbiota communities (Fig. 3C Anosim, p > 0.05). Although the microbiota composition did not have obvious changes between NSCLC patients and non-cancer controls, the taxa with low abundance showed obvious difference. The relative abundances of the taxa were further analyzed to identify taxa-specific differences between NSCLC patients and non-cancer controls in different sampling methods. After removing the species with a very low frequency (positivity < 10%), at the species level, we identified significantly increased bacterial abundance in *Lactobacillus rossiae*, *Burkholderia mallei* and *Bacteroides pyogenes*, et al. and decreased bacterial abundance in *Paenibacillus odorifer*, *Pseudomonas entomophila*, *Magnetospirillum gryphiswaldense* etc. in NSCLC patients compared to non-cancer controls in bronchoscope samples (Fig. 3D).

### Microbiome composition of NSCLC patients and non-cancer controls in lobectomy samples

**Figure 4:**
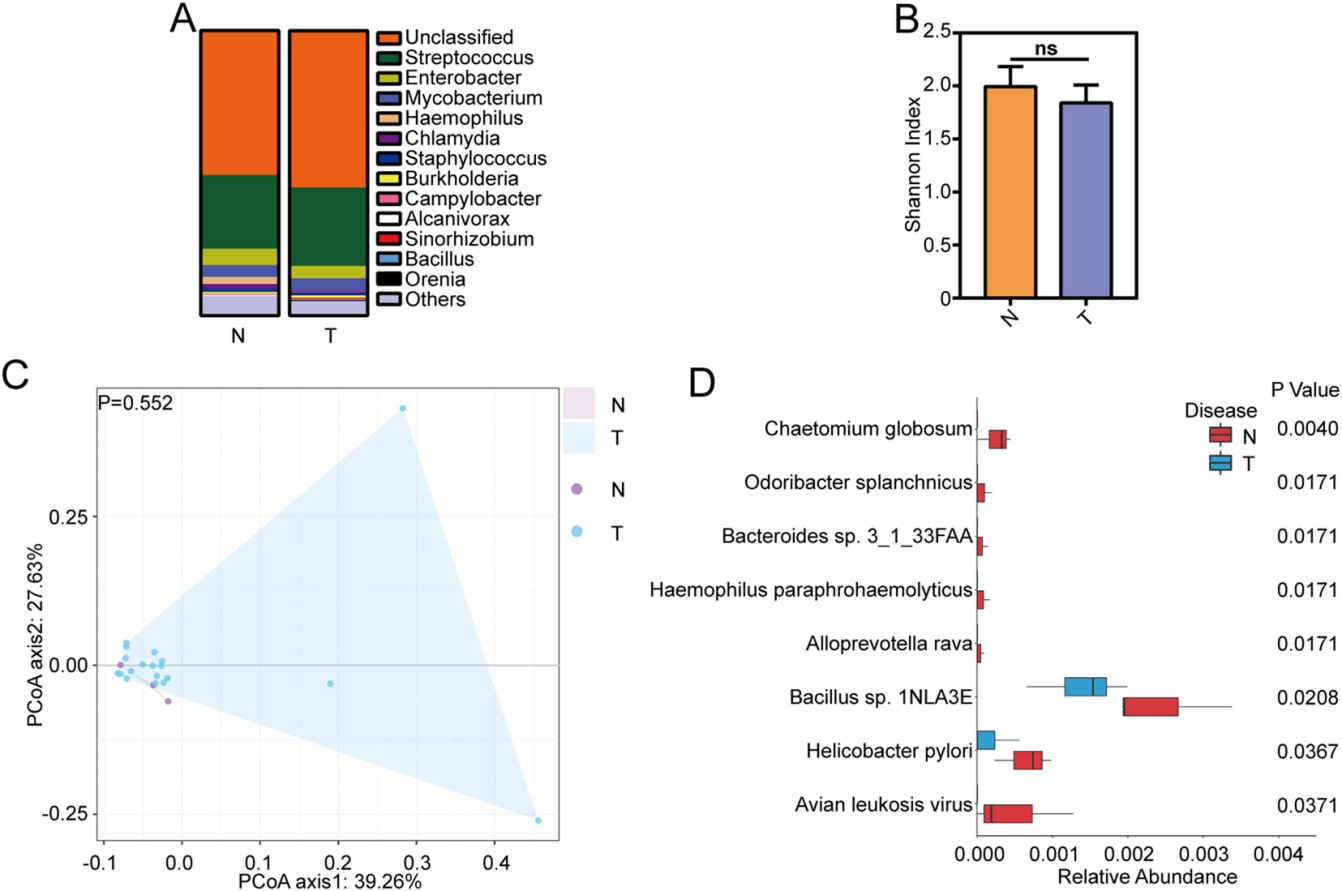
Microbiota composition of NSCLC patients and non-cancer controls in lobectomy samples. (**A**) Taxonomic composition of non-human sequences at phylum level in NSCLC patients and non-cancer controls in lobectomy samples. (**B**) The Shannon diversity index between NSCLC patients and non-cancer controls in lobectomy samples. Difference between sampling methods was determined by Student t test. *NS* no significance. (**C**) PCoA analysis of NSCLC patients and non-cancer controls in lobectomy samples. Anosim was performed to test statistically whether there is a significant difference. p < 0.05 was considered to be significant. (**D**) The differential species between NSCLC patients and non-cancer controls in lobectomy samples were identified by Wilcoxon rank-sum test (p < 0.05). *N* non-cancer controls, *T* NSCLC patients.

In lobectomy samples, the most common genera were *Streptococcus, Enterobacter* and *Mycobacterium* in both NSCLC patients and non-cancer controls, which suggested that the abundance of dominant bacteria did not have obvious difference in NSCLC patients (Fig. 4A). The Shannon diversity index did not show difference between non-cancer controls and NSCLC patients (Fig. 4B). The principal coordinate analysis (PCoA) suggested that NSCLC patients and non-cancer controls had similar microbiota communities no matter what sampling methods have been used (Fig. 4C, Anosim, p > 0.05). Some rare species such as fungus *Chaetomium globosum*, *Alloprevotella rava*, *Haemophilus paraphrohaemolyticus* et al. were found decreased in NSCLC patients compared to that in non-cancer controls. No obvious overpresented bacteria were detected in NSCLC patients for lobectomy samples (Fig. 4D).

### Species in NSCLC patients are age-, gender- and smoking- related and associated with EGFR expression

We then sought to examine the host factors affecting the lung microbiome in NSCLC patients. We analyzed the age-, sex- and smoking-based differences in the lung microbiota composition in these 32 NSCLC patients. The patients older than 60 years old showed higher abundance of *Lactobacillus fabifermentans, Pantoea stewartii, Lactobacillus rossiae *etc*.* and lower abundance of *Klebsiella pneumoniae*, *Prevotella oryzae* compared to patients younger than 60 years (supplementary Fig. 1A). All the age-related species in NSCLC patients were shown in supplementary table 3. The identified species in the male patients and female patients showed differences both in bacterial and fungal species, with higher abundance of *Enterobacter hormaechei*, *Staphylococcus aureus*, and *Talaromyces marneffei *etc*.* in female and higher abundance of *Chromobacterium haemolyticum, Porphyromonas gingivalis *et al*.* in male (supplementary Fig. 1B). All the sex-related species were shown in supplementary table 4. Compared with the NSCLC patients with no smoking history, NSCLC patients with a smoking history showed a higher abundance of *Pseudoalteromonas sp. CF149, Roseburia hominis* and fungus *Penicillium expansum *etc*.* (supplementary Fig. 1C). *Pseudomonas mosselii* and *Pseudomonas putida* were shown decreased in NSCLC patients with a smoking history.

**Figure 5:**
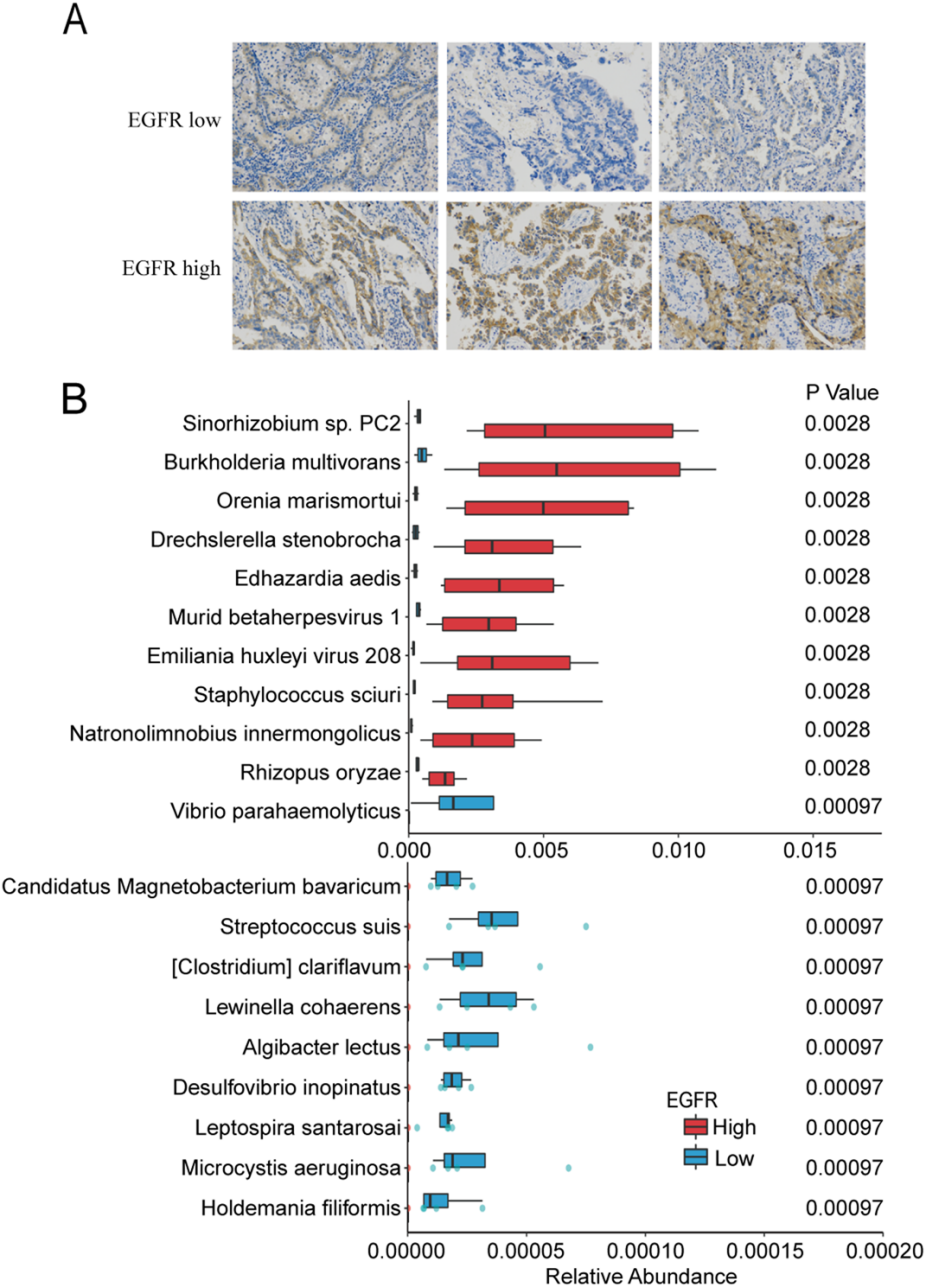
EGFR expression related microbiota composition in NSCLC patients. Representative images of the immunohistochemical detection of EGFR (**A**). Magnification: ×100. (**B**) The differential species in bronchoscopy samples between EGFR high patients and EGFR low patients were identified by Wilcoxon rank-sum test (p < 0.05).

EGFR expression in cancer cells can help define therapy strategies of lung cancer patients. We then measured the expression of EGFR (Fig. 5A) expressions in cancer biopsies from 32 NSCLC patients. In bronchoscopy samples, the ratio of EGFR expression in cancer cells was positively correlated with the abundance of Rhizopus oryzae, Natronolimnobius innermongolicus,*, Staphylococcus sciuri, *etc. (Fig. 5B)*.*

## Discussion

In this study, by using metagnomic sequencing techniques, we found the obvious variation between sampling methods. Oral taxa can be found in both BAL samples, suggesting that the oral microbiota disperse from upper airway to lower airway. The lowest branches of the lungs have distinct microbiota composition from the upper segments, suggesting that the immune system may eliminate the microorganisms that aspirated from oral cavity. Although gross changes of microbiota composition did not occur in NSCLC patients, some rare microbes showed obvious difference, which may indicate the pathogenic roles in the cancer progression.

There is currently no consensus about a healthy lung microbiota^5^. Although there were opinions that healthy lungs lacked resident microbes, it has been reported that in healthy individuals, *Prevotella*, *Streptococcus, Veillonella* and *Neisseria* were the most abundant genera in the lungs^6^. Technical challenges, such as low microbial biomass and bronchoscope contamination, have hindered the identification of specific microbes^18^. Different sampling methods can strongly influence microbiome and provide different readouts and information^19^. BAL samples have been used to characterize the lung microbiome^20,21^. Bronchoscopy is commonly used to collect the BAL. There are 20–25 generations of branches from the trachea to the lung periphery. A bronchoscope can only reach the 4–5th generation of bronchi^22^. Samples collected by bronchoscopy may contains microbes from upper airway. In this study, BAL fluid was collected by two sampling methods from the lower region of the respiratory tract. Bronchoscopic lavage fluid was collected from the upper lobe site and bronchi. We recovered intra-alveoli lavage which represents the lowest branches of respiratory tract. We found that the lung microbiota displayed great spatial variation between sites in the lung. However, the microbiota from both sampling methods were enriched with oral-related taxa, such as *Streptococcus, Enterobacter,* indicating that the detection of oral microbiota and upper airway in bronchoalveolar lavage are most likely the result of aspiration of oral secretions and oral microorganisms are true members of the lung microbiota^9^. The dominant genus was shifted from *Neisseria* to *Mycobacterium* in lobectomy samples*,* indicating that the lowest branches of airway have different environment suitable for specific bacteria.

Compared to the obvious variable microbial composition between anatomy sites, the microbiota composition did not show obvious difference between NSCLC patients and non-cancer controls. It suggested that only a small percentage of the variance in lung microbiome in lung cancer patients. Undoubtedly, microbiome-derived products that target dysbiosis have brought exciting potential to many medical challenges. However, the problems confronting dysbiosis correction are manifold^23^. No consensus on a definition of “dysbiosis” , small sample size, confounding factors and different manifestations of the same disease make the discrepancy between studies^23^. The case–control studies are statistically underpowered because of small sample size. In this study, a relatively small sample size (n = 47) may affect the statistical power. A meta-analysis may increase the power of small studies^23^. In a meta-analysis with a big sample size revealed only marginal differences for microbiota diversity in obese individuals which is different from study with smaller sample size^24^. In advanced lung cancer, lower airway dysbiosis is associated with poor prognosis^16^. Some studies attributed microbiota changes into three distinct categories. One category is characterized by an enrichment of a small number of potential pathogens. Another category is characterized by a depletion of probiotics. Gross changes of microbiota composition also happened in some diseases^23^. In this study, lung cancer did not cause marked shifts in the principal component analysis space of microbiota composition. Low-abundance bacteria such as *Bacteroides pyogenes*, *lactobacillus rossiae* and *Burkholderia mallie* were enriched in NSCLC patients, suggesting their potential pathogenesis. We also found the depletion of some bacteria such as *Chaetomium globosum*, *Odoribacter splanchnicus* et al. The roles of these specific species need to be further studied. The identification of microbial biomass specimens from liquid samples and mucus surface is more challenging than stool due to low density and high host DNA contamination^25^. The mapping rates in this study showed that more than 50% and as much as approximately 95% of DNA was from human nucleotide fractions. Culture techniques, especially fungal culture techniques, are limited. Unculturable bacteria and fungi comprise the largest part of the human microbiota^26^. Culture-independent molecular approaches provide a sensitive and specific technique to detect microorganisms. In this study we employed the shotgun metagenomic sequencing, which uses random primers to sequence sheared DNA fragments, to characterize the bacterial, fungal and viral communities in the lungs. Metagenomic sequencing provides an approach to species-level analysis for low-abundance organisms. For example, shotgun-sequencing efforts have suggested that fungi account for approximately 0.1% of the microorganisms^27^. The shotgun sequencing is also superior to 16s sequencing for avoiding the contamination during PCR procedures. By using metagenomic shotgun sequencing, we found that *Streptococcus, Enterobacter* and *Mycobacterium* were dominant in the lungs across 22 lobectomy samples. The numerical dominance of these microbes (always > 1%) implies that they are compatible with the host immune system^28^. We found that *Mycobacterium tuberculosis* was obviously overpresented in lobectomy samples than in bronchoscope samples. *Mycobacterium tuberculosis* has the extraordinary ability to persist for decades. The pathogenesis of *Mycobacterium tuberculosis* is associated with host–pathogen interactions^29^. However, the prevalence of *Mycobacterium tuberculosis* may represent regional bias. The roles of *Mycobacterium tuberculosis* in regional epidemiology need to be further investigated. The decreased bacteria is most likely due to elimination by the immune system and immigration from the mouth and upper airways^5,30^. The maintenance of the lung microbiota depends on factors such as oxygen pressure, temperature or the presence of secretory molecules^31–33^.

The lung microbiota can be influenced by the environment. Genera *Vibrio*, *Enterobacter*, and *Escherichia* which are not considered as members of the lung microbiota were detected in our study. There are many gut microbial species such as *Escherichia coli* that are found in the lungs. *Escherichia coli* can be translocated to the lungs by aspiration, which is referred to as fecal–oral transmission, or can be translocated in the immune cells via the bloodstream^5^.

Lung diseases alter the microbiota composition, showing a shift in the lung microbiota. *Streptococcus pneumoniae, Streptococcus aureus, Haemophilus influenza, Escherichia coli, Pseudomonas aeruginosa* and *Moraxella caterrhalis* were considered to be the principal pathogens for lung cancer patients with clinical infection symptoms^34^. However, the role of these pathogens in cancer progression has not been determined. There have been reports that link more abundance of genera *Veillonella* and *Megasphaera* with lung cancer^35^. A pilot study on the sputum samples demonstrated significantly higher abundance of *Granulicatella adiacens*, *Streptococcus intermedius* and *Mycobacterium tuberculosis* in lung cancer patients^36^. In the 47 individuals enrolled in this case–control study, a lower abundance of *Chaetomium globosum, Odoribacter splanchnicus, Bacteroides spp.,Haemophilus paraphrohaemolyticus, Appoprevotella rava, *etc., were detected in NSCLC patients compared to non-cancer controls. *Lactobacillus rossiae*, *Burkholderia mallei*, *Bacteroides pyogenes* etc. were found to be higher in NSCLC patients compared to non-cancer controls. Unexpectedly, *lactobacillus rossiae,* which is considered to be probiotics in intestinal microbiota was observed increased in NSCLC patients and the patients older than 60 years old. *Lactobacillus rossiae* was reported to have the ability to de novo production of vitamin B12^37^. The side effects of vitamin B supplementation has been reported and Vitamin B12 was linked with the increased risk of lung cancer^38^. *Lactobacillus salivarius* has been found increased in metastatic lung cancer patients^39^. The roles of these rare species in lung cancer progression remain to be elucidated.

Lung cancer in never smokers and females accounts for approximately 25% lung cancer patients worldwide. These subtypes of patients display distinctive clinical characteristics and good response to EGFR-TKI. The differential species between males and females, smokers and non-smokers, for example *Talaromyces marneffei* may hint at the benefits of EGFR-TKI therapy efficacy. Further research needs to be done to figure out how specific bacteria can inhibit the lung cancer progression and what specific bacteria can be prescribed as an adjuvant therapy for patients.

Although a huge potential in lung cancer research, microbiome research meets problems of reproducibility and data synthesis across studies^40^. The lung microbiome composition is highly dynamic and diverse because of environmental exposures, antibiotics exposure, disease process or microbial community interactions et al. In case–control studies of the human microbiome, although multiple risk factors can be evaluated, there are still challenges in the analysis of microbial community composition and rare taxa detection^41^. One limitation of this study is that we cannot consider the impact of diet, geographical locations and other lifestyle factors. A workflow of shotgun metagenomic data includes quality trimming and matching the sequencing reads onto a reference database. Interference of DNA contamination including host DNA and environmental DNA is a problem for shotgun metagenomic sequencing. In this study, the samples were collected from the same hospital under sterile operating room to reduce the contamination. Because some taxa detected may represent contamination of samples, some research applied filters to eliminate contamination. However, how to do with sequencing data from “background” is still under debate. Simply removing all of the bacterial OTUs found in background is not appropriate^42^. The presence of contamination can be determined by using a cutoff based on the number of sequences^41^. Several methods have been suggested to remove contaminating DNA from reagents and the environment, including UV and gamma radiation^42^. Negative control samples including DNA extraction controls, PCR controls and paraffin controls were used to be background and bacterial species with abundance bigger than 7.5% in negative controls were removed from results^43^. The BAL samples were collected and stored in sterile plasticwares in this study. The sampling methods reduced most contamination and the results represented the species commonly seen in lower airway. In this study, about 50% of species were “unclassified” and species with abundance bigger than 10% was *Streptococcus pneumoniae,* which is considered to be the member of lung microbiota. Despite that, the inclusion of background is still recommended.

## Methods

### Study population and sample selection

For metagenomic sequencing, BAL were obtained from 47 individuals, including 15 non-cancer controls (patients with benign pulmonary diseases) and 32 NSCLC patients from 2019 to 2020.All cases enrolled in this study and surgical resection specimens were collected at the second Xiangya hospital, Central South University, China. The clinical characteristics of the subjects are summarized in Table 1. These patients underwent bronchoscopy or lobectomy for diagnosis or therapy. There were no adverse clinical events related to sampling. We collected the BAL from bronchoscope aspirate and alveolar lavage after lobectomy of lung without passing through the upper airway and bronchioles. Twenty-five samples were obtained under sterile conditions by instillation and aspiration of 20 ml of 0.9% NaCl from the bronchoscope. For the lower bronchi, we collected intra-alveolar BAL by instillation and aspiration of 20 ml of 0.9% NaCl from lung resection specimens. All samples were collected in sterile conditions. These patients had not been treated with antibiotics. Patients with clinical evidence of infection, sepsis or active tuberculosis were excluded. The patients were informed of the sample collection and signed informed consent forms. The collection and use of samples were approved by the ethical review committees of the second Xiangya Hospital, Central South University. The BAL samples were frozen in sterile containers and stored at − 80 °C before DNA extraction. The research presented here has been performed in accordance with the Declaration of Helsinki and has been approved by the ethics committee of the Second Xiangya Hospital, Central South University, China. The patients were informed about the sample collection and had signed informed consent forms.

### Metagenomic sequencing

The metagenomic sequencing was performed by the paired-end sequencing method on the Illumina platform (BGI, China). Briefly, DNA was sheared by ultrasonication (Covaris, Woburn, MA). The sheared DNA fragments were end-repaired (DNA End Repair Mix) at 20 °C for 30 min. The DNA fragments were purified by QIAquick PCR Purification Kit (Qiagen) and A-tailed using A-Tailing Mix. Libraries were checked using Bioanalyzer 2100 (Agilent) and quantified using the ABI StepOnePlus Real-Time PCR System. Libraries were sequenced on an Illumina platform. The contigs obtained after de novo assembly were applied for gene prediction using MetaGeneMark. The predicted genes were clustered using CD-hit. The reads were mapped to this combined gene pool using Bowtie 2 for quantification of genes and species. All the analyses were conducted by BGI, China.

### Immunohistochemistry

Tumor tissues were fixed and embedded in paraffin wax. After being dewaxed and rehydrated,the slides were dipped into EDTA buffer (1 mM EDTA, 0.05% Tween 20, pH8.0) followed by microwave heating to recoverantigen. Tissue sections wereincubated with primary EGFR antibody (RMA-0689, Maixin, China) at 4 °C overnight. After washing with PBS, sections were incubated with biotinylated goat anti-rabbit IgG antibodies (UltraSensitive S-P Kit, Maixin Biotechnology, China). The sections were then washed and incubated with an avidin–biotin complex for 1 h. After washing with PBS,the sections were treated with 3′-diaminobenzidine hydrochloride (DAB) and were counterstained with hematoxylin. The sections were observed and imaged under a microscope (NIKON, Japan). DEPC (Diethyl pyrocarbonate) water was used instead of the first antibody as a negative control.

### Statistical analysis

Species gene frequency profiles were established by matching the sequencing reads from an individual sample onto a reference catalogue. Correlation between bronchoscope and lobectomy samples or NSCLC and Non-cancer control samples was analyzed by NMDS and PCoA. Neutral model with the bronchoscope samples was used as a source of OTUs found in the lobectomy samples to evaluate if microbial community in lungs is region specific. The Sloan neutral model is fit to the observed frequency of occurrence of OTUs using R. 95% confidence intervals around prediction model were calculated with Wilson score interval in R. The differential genes between bronchoscope and lobectomy samples were identified using the Wilcoxon rank-sum test after neutral model. The NSCLC-, age-, gender-, smoking history- and EGFR expression-based difference in abundance of species were determined by Wilcoxon rank-sum test. A value of p < 0.05 was considered statistically significant. Biostatistical analysis was implemented based on package “vegan” using the R statistical computing environment (R version 4.0, 2020, https://www.R-project.org). Diversity indices were assessed by calculating the Shannon diversity index. Differences between the two groups were determined by the Student’s t test. A value of p < 0.05 was considered statistically significant.

### Ethical approval

The research presented here has been performed in accordance with the Declaration of Helsinki and has been approved by the ethics committee of the Second Xiangya Hospital, Central South University, China. The patients were informed about the sample collection and had signed informed consent forms.

## Supplementary Information

Supplementary Information 1.

Supplementary Information 2.

## Data Availability

All the data generated or analyzed during this study are included in this published article and its supplementary files. The metagenomic sequencing data presented in this study can be found in NCBI Sequence Read Archive (SRA) database. The accession number(s) is SRA, PRJNA714488 and can be accessible with the following link: https://www.ncbi.nlm.nih.gov/sra/PRJNA714488.
