# Supplementary material for: Lung microbiome alterations in NSCLC patients
Source: Sci Rep. 2021 Jun 3;11:11736. doi: 10.1038/s41598-021-91195-2 (PMC8175694; doi:10.1038/s41598-021-91195-2)
Supplement: Supplementary file 1 — Supplementary Information 1. [file 41598_2021_91195_MOESM1_ESM.docx]

Lung microbiome alterations in NSCLC patients

Leliang Zheng^#1,2,6,7^, Ruizheng Sun^#1,2,6,7^, Yinghong Zhu^1,2,6,7^, Zheng Li^1,2,6,7^， Xiaoling She^5^, Xingxing Jian^1,2,6,7^, Fenglei Yu^3^, Xueyu Deng^3^, Buqing Sai^1,2,6,7^, Lujuan Wang^1,2,6,7^, Wen Zhou^1,2,6,7^, Minghua Wu^1,2,6,7^, Guiyuan Li^1,2,6,7^, Jingqun Tang^3^*, Wei Jia^4^*,Juanjuan Xiang^1,2,6,7^*

^1^Hunan Cancer Hospital, the Affiliated Cancer Hospital of Xiangya School of Medicine, Central South University, Changsha, Hunan, PR China

^2^Cancer Research Institute, School of Basic Medical Science, Central South University, Changsha, Hunan, China

^3^Department of thoracic surgery, the Second Xiangya Hospital, Central South University, Changsha, Hunan 410013, China

^4^Hong Kong Phenome Research Centre, School of Chinese Medicine, Hong Kong Baptist University, Kowloon Tong, Hong Kong, China

^5^Department of pathology, the Second Xiangya Hospital, Central South University, Changsha, Hunan 410013, China

^6^NHC Key Laboratory of Carcinogenesis and the Key Laboratory of Carcinogenesis and Cancer Invasion of the Chinese Ministry of Education, Xiangya Hospital, Central South University, Changsha, Hunan, China.

^7^Hunan Key Laboratory of Nonresolving Inflammation and Cancer, Changsha, Hunan, 410013, China

# These authors contributed equally to the manuscript

*Corresponding authors

Prof. Juanjuan Xiang, [xiangjj@csu.edu.cn](mailto:xiangjj@csu.edu.cn)

Prof. Wei Jia, weijia1@hkbu.edu.hk

Prof. Jingqun Tang, tangjq@csu.edu.cn

Running title: Lung microbiome in NSCLC patients

Supplementary figure 1：Age-, gender- and smoking- related microbiota composition in NSCLC patients.


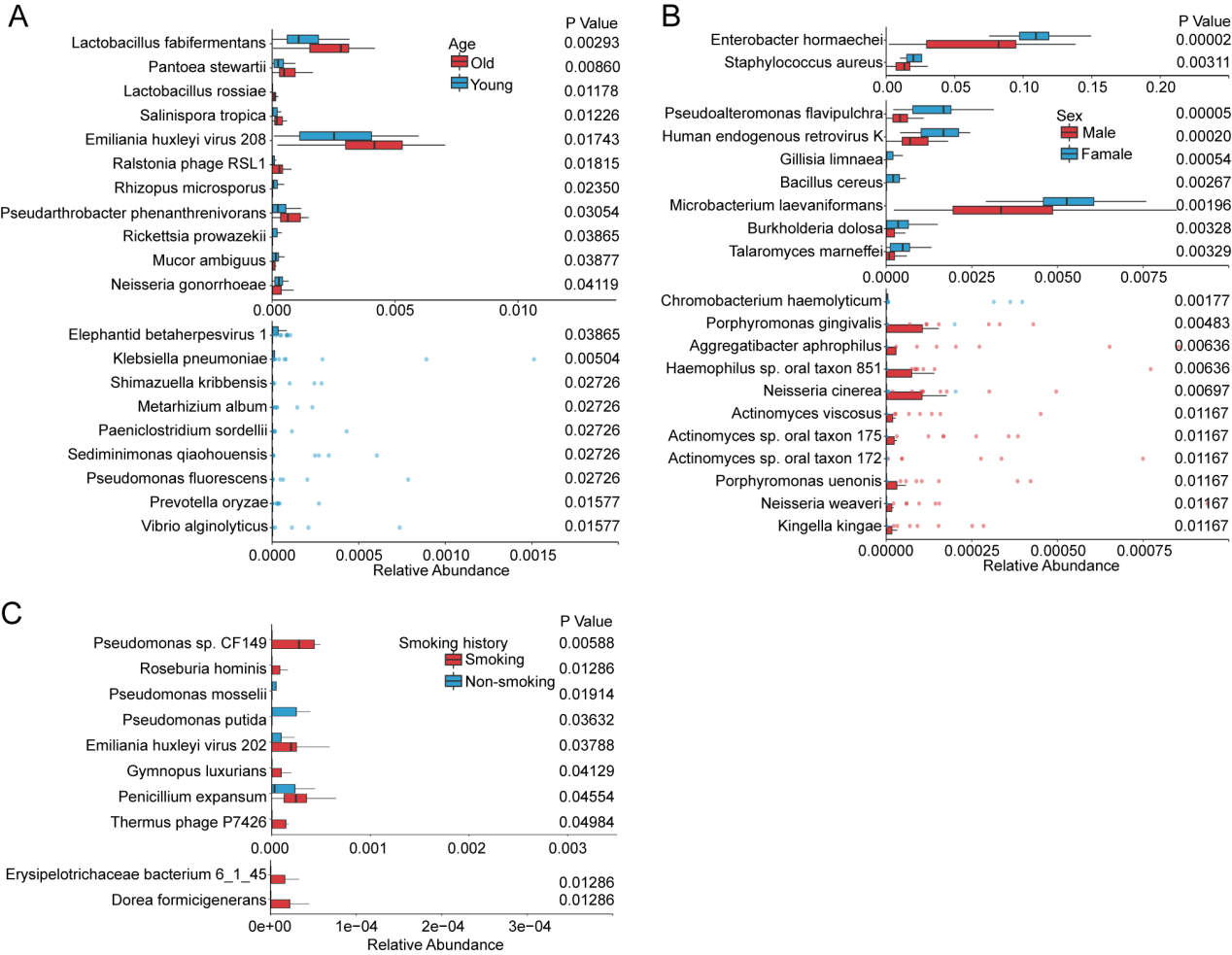


Supplementary figure 1: Age-, gender- and smoking- related microbiota composition in NSCLC patients. The differential abundance of microbiota was determined by Wilcoxon rank-sum test. Taxonomic composition of microbiota in (A) older than 50 years and younger than 50 years; (B) male and female; (C) patients with or without smoking history.
