# Supplementary material for: Lung microbiome alterations in NSCLC patients
Source: Sci Rep. 2021 Jun 3;11:11736. doi: 10.1038/s41598-021-91195-2 (PMC8175694; doi:10.1038/s41598-021-91195-2)
Supplement: Supplementary file 2 — Supplementary Information 2. [file 41598_2021_91195_MOESM2_ESM.docx]

Lung microbiome alterations in NSCLC patients

Leliang Zheng^#1,2,6,7^, Ruizheng Sun^#1,2,6,7^, Yinghong Zhu^1,2,6,7^, Zheng Li^1,2,6,7^， Xiaoling She^5^, Xingxing Jian^1,2,6,7^, Fenglei Yu^3^, Xueyu Deng^3^, Buqing Sai^1,2,6,7^, Lujuan Wang^1,2,6,7^, Wen Zhou^1,2,6,7^, Minghua Wu^1,2,6,7^, Guiyuan Li^1,2,6,7^, Jingqun Tang^3^*, Wei Jia^4^*,Juanjuan Xiang^1,2,6,7^*

^1^Hunan Cancer Hospital, the Affiliated Cancer Hospital of Xiangya School of Medicine, Central South University, Changsha, Hunan, PR China

^2^Cancer Research Institute, School of Basic Medical Science, Central South University, Changsha, Hunan, China

^3^Department of thoracic surgery, the Second Xiangya Hospital, Central South University, Changsha, Hunan 410013, China

^4^Hong Kong Phenome Research Centre, School of Chinese Medicine, Hong Kong Baptist University, Kowloon Tong, Hong Kong, China

^5^Department of pathology, the Second Xiangya Hospital, Central South University, Changsha, Hunan 410013, China

^6^NHC Key Laboratory of Carcinogenesis and the Key Laboratory of Carcinogenesis and Cancer Invasion of the Chinese Ministry of Education, Xiangya Hospital, Central South University, Changsha, Hunan, China.

^7^Hunan Key Laboratory of Nonresolving Inflammation and Cancer, Changsha, Hunan, 410013, China

# These authors contributed equally to the manuscript

*Corresponding authors

Prof. Juanjuan Xiang, [xiangjj@csu.edu.cn](mailto:xiangjj@csu.edu.cn)

Prof. Wei Jia, weijia1@hkbu.edu.hk

Prof. Jingqun Tang, tangjq@csu.edu.cn

Running title: Lung microbiome in NSCLC patients

Supplementary table 1： Difference genera in different sampling methods

|  | BLpvalue | LBratio | trend | Neutral |
| --- | --- | --- | --- | --- |
| Porphyromonas | 9.46E-08 | 0.002856 | down | down |
| Veillonella | 1.17E-07 | 0.002168 | down | down |
| Fusobacterium | 1.43E-07 | 0.000256 | down | down |
| Prevotella | 4.50E-07 | 0.015924 | down | down |
| Alloprevotella | 1.16E-06 | 0.006428 | down | down |
| Morococcus | 2.68E-06 | 0.01323 | down | down |
| Rothia | 3.85E-06 | 0.001807 | down | down |
| Parabacteroides | 5.09E-06 | 0.006665 | down | down |
| Porphyrobacter | 1.39E-05 | 0.025992 | down | down |
| Actinomyces | 2.26E-05 | 5.56E-05 | down | down |
| Capnocytophaga | 2.26E-05 | 0.002515 | down | down |
| Aggregatibacter | 3.24E-05 | 0.065916 | down | down |
| Selenomonas | 5.66E-05 | 0.000104 | down | down |
| Mycobacterium | 6.20E-05 | 1.507804 | up | up |
| Kingella | 0.00101817 | 0.007144 | down | down |
| Devosia | 0.001410356 | 1.876595 | up | up |
| Pseudoalteromonas | 0.00148404 | 2.065148 | up | up |
| Streptococcus | 0.001742941 | 1.52691 | up | up |
| Enterococcus | 0.001747706 | 0.013258 | down | down |
| Pseudarthrobacter | 0.002211042 | 2.535689 | up | up |
| Sinorhizobium | 0.002576228 | 1.745841 | up | up |
| Orenia | 0.002576228 | 1.735001 | up | up |
| Erysiphe | 0.002780369 | 1.818124 | up | up |
| Jiangella | 0.003321881 | 2.390833 | up | up |
| Drechslerella | 0.003482159 | 1.886008 | up | up |
| Natronolimnobius | 0.004661143 | 1.848438 | up | up |
| Talaromyces | 0.004876762 | 1.866562 | up | up |
| Bacteroides | 0.005684784 | 0.118299 | down | down |
| Paenibacillus | 0.00576467 | 1.552061 | up | up |
| Hungatella | 0.00634949 | 0.126189 | down | down |
| Rodentibacter | 0.007080036 | 0.591319 | down | down |
| Alicyclobacillus | 0.007944354 | 1.753611 | up | up |
| Pasteurella | 0.008217965 | 0.627052 | down | down |
| Anabaena | 0.00914809 | 0.020152 | down | down |
| Edhazardia | 0.010573243 | 1.654635 | up | up |
| Desulfitobacterium | 0.011832353 | 0.09507 | down | down |
| Muromegalovirus | 0.01202532 | 1.67658 | up | up |
| Salinispora | 0.014526872 | 1.591521 | up | up |
| Achromobacter | 0.015455084 | 0.004689 | down | down |
| Coccolithovirus | 0.01643364 | 1.59865 | up | up |
| Verminephrobacter | 0.01643364 | 1.756232 | up | up |
| Saccharomyces | 0.020127906 | 1.672713 | up | up |
| Chlamydia | 0.020899341 | 1.177423 | up | up |
| Lodderomyces | 0.026361323 | 1.787794 | up | up |
| Ophiocordyceps | 0.027901547 | 1.407368 | up | up |
| Ruminococcus | 0.029651842 | 1.633934 | up | up |
| Cardiobacterium | 0.030372272 | 0.019505 | down | down |
| Bacillus | 0.031210574 | 1.317579 | up | up |
| Rhizophagus | 0.036484677 | 1.356316 | up | up |

Supplementary table 2: Difference species in different sampling methods

| [Species](javascript:;) | BLpvalue | LBratio | trend | Neutral |
| --- | --- | --- | --- | --- |
| Porphyromonas somerae | 4.44E-08 | 0.000422 | down | down |
| Porphyromonas endodontalis | 1.40E-07 | 0.004041 | down | down |
| Fusobacterium periodonticum | 1.43E-07 | 0.000243 | down | down |
| Prevotella intermedia | 1.50E-07 | 0.003617 | down | down |
| Prevotella nanceiensis | 2.56E-07 | 0.003214 | down | down |
| Prevotella aurantiaca | 4.94E-07 | 0.000629 | down | down |
| Alloprevotella tannerae | 6.01E-07 | 0.005711 | down | down |
| Haemophilus parainfluenzae | 9.31E-07 | 0.035448 | down | down |
| Prevotella melaninogenica | 9.99E-07 | 0.006278 | down | down |
| Porphyromonas sp. KLE 1280 | 1.12E-06 | 0.004676 | down | down |
| Neisseria lactamica | 1.13E-06 | 0.001973 | down | down |
| Streptococcus mitis | 1.60E-06 | 0.01745 | down | down |
| Neisseria mucosa | 2.40E-06 | 0.002555 | down | down |
| Prevotella sp. oral taxon 473 | 2.44E-06 | 0.004533 | down | down |
| Veillonella sp. oral taxon 158 | 2.74E-06 | 0.002932 | down | down |
| Morococcus cerebrosus | 3.00E-06 | 0.011183 | down | down |
| Porphyromonas sp. oral taxon 279 | 3.21E-06 | 0.000516 | down | down |
| Neisseria sicca | 3.44E-06 | 0.004381 | down | down |
| Neisseria flavescens | 6.09E-06 | 0.005034 | down | down |
| Neisseria meningitidis | 6.47E-06 | 0.006091 | down | down |
| Veillonella dispar | 8.68E-06 | 0.000329 | down | down |
| Prevotella sp. C561 | 8.68E-06 | 0.000106 | down | down |
| Prevotella sp. oral taxon 299 | 8.68E-06 | 0.000512 | down | down |
| Streptococcus pseudopneumoniae | 8.99E-06 | 0.00594 | down | down |
| Porphyrobacter donghaensis | 1.25E-05 | 0.022257 | down | down |
| Capnocytophaga sp. oral taxon 329 | 2.52E-05 | 0.000456 | down | down |
| Rothia mucilaginosa | 2.80E-05 | 0.00197 | down | down |
| Neisseria macacae | 2.80E-05 | 0.012973 | down | down |
| Prevotella marshii | 4.26E-05 | 0.022112 | down | down |
| Bacteroides sp. 2_1_56FAA | 4.33E-05 | 0.032638 | down | down |
| Neisseria cinerea | 5.66E-05 | 0.000477 | down | down |
| Aggregatibacter sp. oral taxon 458 | 5.79E-05 | 0.020689 | down | down |
| Prevotella histicola | 6.30E-05 | 0.000378 | down | down |
| Neisseria elongata | 7.01E-05 | 0.001175 | down | down |
| Neisseria sp. GT4A_CT1 | 7.30E-05 | 0.013437 | down | down |
| Prevotella pallens | 7.48E-05 | 0.013718 | down | down |
| Alloprevotella rava | 7.79E-05 | 0.01628 | down | down |
| Prevotella salivae | 7.79E-05 | 0.017387 | down | down |
| Mycobacterium tuberculosis | 0.000116 | 1.440137 | up | up |
| Prevotella disiens | 0.000137 | 0.000401 | down | down |
| Neisseria sp. oral taxon 014 | 0.000152 | 0.00073 | down | down |
| Prevotella enoeca | 0.000152 | 0.004202 | down | down |
| uncultured prokaryote | 0.00028 | 0.035106 | down | down |
| Veillonella parvula | 0.00032 | 0.000907 | down | down |
| Neisseria sp. oral taxon 020 | 0.00032 | 0.000569 | down | down |
| Porphyromonas gingivalis | 0.000354 | 0.000156 | down | down |
| Prevotella bivia | 0.000384 | 0.047199 | down | down |
| Aggregatibacter segnis | 0.000498 | 0.13822 | down | down |
| Haemophilus haemolyticus | 0.000546 | 0.533113 | down | down |
| Bacteroides ovatus | 0.000823 | 0.058589 | down | down |
| Prevotella buccae | 0.000853 | 0.120397 | down | down |
| Bacteroides oleiciplenus | 0.000853 | 0.0692 | down | down |
| Haemophilus pittmaniae | 0.000938 | 0.144265 | down | down |
| Parabacteroides distasonis | 0.001068 | 0.016041 | down | down |
| Pseudomonas putida | 0.001221 | 0.010496 | down | down |
| Pseudoalteromonas flavipulchra | 0.001368 | 2.197927 | up | up |
| Selenomonas sp. CM52 | 0.001589 | 0.000231 | down | down |
| Sinorhizobium sp. PC2 | 0.001609 | 1.66726 | up | up |
| Paenibacillus odorifer | 0.001609 | 2.148016 | up | up |
| Burkholderia pseudomallei | 0.002208 | 1.732369 | up | up |
| Bacteroides vulgatus | 0.00228 | 0.044977 | down | down |
| Shigella dysenteriae | 0.002538 | 0.008734 | down | down |
| Drechslerella stenobrocha | 0.002576 | 1.805365 | up | up |
| Chlamydia psittaci | 0.002576 | 1.704939 | up | up |
| Prevotella multiformis | 0.002781 | 0.064003 | down | down |
| Bacteroides coprophilus | 0.002781 | 0.017028 | down | down |
| Prevotella copri | 0.003173 | 0.216198 | down | down |
| Shigella flexneri | 0.003274 | 0.028094 | down | down |
| Orenia marismortui | 0.003482 | 1.645473 | up | up |
| Aggregatibacter actinomycetemcomitans | 0.003644 | 0.145739 | down | down |
| Prevotella timonensis | 0.003713 | 0.00145 | down | down |
| Porphyromonas macacae | 0.003713 | 0.003086 | down | down |
| Devosia chinhatensis | 0.003744 | 1.908933 | up | up |
| Erysiphe necator | 0.004034 | 1.737647 | up | up |
| Pseudarthrobacter phenanthrenivorans | 0.004413 | 2.332124 | up | up |
| Streptococcus pneumoniae | 0.004661 | 1.486835 | up | up |
| Prevotella oris | 0.004881 | 0.05889 | down | down |
| Jiangella alkaliphila | 0.005685 | 2.201134 | up | up |
| Natronolimnobius innermongolicus | 0.006181 | 1.773855 | up | up |
| Hungatella hathewayi | 0.006349 | 0.119567 | down | down |
| Bacteroides fragilis | 0.006481 | 0.138079 | down | down |
| Rodentibacter pneumotropicus | 0.006579 | 0.565793 | down | down |
| Porphyromonas gulae | 0.007021 | 0.002382 | down | down |
| Kingella oralis | 0.007675 | 0.000612 | down | down |
| Streptococcus parasanguinis | 0.007675 | 0.003708 | down | down |
| [Enterobacter] aerogenes | 0.008008 | 4.207439 | up | up |
| Edhazardia aedis | 0.011279 | 1.576179 | up | up |
| Staphylococcus sciuri | 0.011279 | 1.524359 | up | up |
| Bacteroides uniformis | 0.011832 | 0.049609 | down | down |
| Alicyclobacillus ferrooxydans | 0.012239 | 1.660219 | up | up |
| Prevotella bryantii | 0.01399 | 0.341992 | down | down |
| Enterococcus faecalis | 0.014184 | 0.002177 | down | down |
| Streptococcus oralis | 0.014312 | 0.338699 | down | down |
| Streptococcus sp. GMD4S | 0.015455 | 0.007817 | down | down |
| Anabaena sp. 90 | 0.016826 | 0.012094 | down | down |
| Burkholderia cenocepacia | 0.016826 | 0.034457 | down | down |
| Ruminococcus callidus | 0.017952 | 1.677455 | up | up |
| Pseudomonas entomophila | 0.018302 | 0.037373 | down | down |
| Talaromyces marneffei | 0.018797 | 2.229684 | up | up |
| Emiliania huxleyi virus 208 | 0.022165 | 1.560051 | up | up |
| Bacteroides massiliensis | 0.025108 | 0.270969 | down | down |
| Murid betaherpesvirus 1 | 0.026361 | 1.590274 | up | up |
| Verminephrobacter aporrectodeae | 0.026361 | 1.66028 | up | up |
| Porphyromonas gingivicanis | 0.027966 | 0.002393 | down | down |
| Prevotella sp. MSX73 | 0.027971 | 0.173001 | down | down |
| Prevotella conceptionensis | 0.030085 | 0.202732 | down | down |
| Achromobacter xylosoxidans | 0.030372 | 0.004308 | down | down |
| Streptococcus anginosus | 0.030372 | 0.00454 | down | down |
| Phaeocystis globosa virus 12T | 0.034794 | 1.569301 | up | up |
| Lactobacillus rhamnosus | 0.035722 | 0.082191 | down | down |
| Lodderomyces elongisporus | 0.04095 | 1.682492 | up | up |
| Rhizophagus irregularis | 0.042615 | 1.393652 | up | up |
| uncultured bacterium | 0.043173 | 1.439894 | up | up |
| Burkholderia dolosa | 0.04466 | 2.335461 | up | up |
| Haemophilus sp. oral taxon 851 | 0.045241 | 0.351373 | down | down |
| Haemophilus paraphrohaemolyticus | 0.045241 | 0.299357 | down | down |
| Ophiocordyceps sinensis | 0.04792 | 1.330469 | up | up |
| Bacillus sp. 1NLA3E | 0.04792 | 1.47726 | up | up |

Supplementary table 3: Age-related species in NSCLC patients

| species | pvalue | Old-Young-ratio | trend |
| --- | --- | --- | --- |
| Lactobacillus fabifermentans | 0.002928 | 1.821392 | up |
| Klebsiella pneumoniae | 0.005045 | 0 | down |
| Pantoea stewartii | 0.008597 | 1.979503 | up |
| Lactobacillus rossiae | 0.011777 | 3.884219 | up |
| Salinispora tropica | 0.012262 | 2.390517 | up |
| Vibrio alginolyticus | 0.015769 | 0 | down |
| Prevotella oryzae | 0.015769 | 0 | down |
| Emiliania huxleyi virus 208 | 0.017429 | 1.473903 | up |
| Ralstonia phage RSL1 | 0.018145 | 2.968697 | up |
| Rhizopus microsporus | 0.023497 | 0.091656 | down |
| Pseudomonas fluorescens | 0.027261 | 0 | down |
| Sediminimonas qiaohouensis | 0.027261 | 0 | down |
| Paeniclostridium sordellii | 0.027261 | 0 | down |
| Metarhizium album | 0.027261 | 0 | down |
| Shimazuella kribbensis | 0.027261 | 0 | down |
| Pseudarthrobacter phenanthrenivorans | 0.030538 | 1.65123 | up |
| Rickettsia prowazekii | 0.038652 | 0.169983 | down |
| Elephantid betaherpesvirus 1 | 0.038652 | 0.19819 | down |
| Mucor ambiguus | 0.038765 | 0.460269 | down |
| Neisseria gonorrhoeae | 0.041194 | 0.38273 | down |
| Pseudomonas entomophila | 0.041696 | 0.056651 | down |
| Vibrio harveyi | 0.046603 | 0 | down |
| Rhodococcus phage ReqiPepy6 | 0.046603 | 0 | down |
| Arenimonas oryziterrae | 0.046603 | 0 | down |
| Pseudoflavonifractor capillosus | 0.046603 | 0 | down |
| Syntrophomonas wolfei | 0.046603 | 0 | down |
| Pseudomonas sp. CT14 | 0.046603 | 0 | down |
| Sinorhizobium sp. CCBAU 05631 | 0.047704 | 0.451764 | down |

Supplementary table 4: Sex-related species in NSCLC patients

| species | pvalue | Male-female-ratio | trend |
| --- | --- | --- | --- |
| Enterobacter hormaechei | 2.07E-05 | 0.495985 | down |
| Pseudoalteromonas flavipulchra | 5.13E-05 | 0.327688 | down |
| Human endogenous retrovirus K | 0.000197 | 0.409769 | down |
| Gillisia limnaea | 0.000539 | 0.037965 | down |
| Chromobacterium haemolyticum | 0.00177 | 0 | down |
| Microbacterium laevaniformans | 0.001957 | 0.213988 | down |
| Bacillus cereus | 0.00267 | 0.690245 | down |
| Staphylococcus aureus | 0.003109 | 0.5352 | down |
| Burkholderia dolosa | 0.00328 | 0.376897 | down |
| Talaromyces marneffei | 0.003287 | 0.385977 | down |
| Chlamydia trachomatis | 0.004497 | 0.670222 | down |
| Porphyromonas gingivalis | 0.004834 | 104.4299 | up |
| Clostridiales bacterium 1_7_47FAA | 0.005977 | 0.639908 | down |
| Aggregatibacter aphrophilus | 0.006359 | Inf | up |
| Haemophilus sp. oral taxon 851 | 0.006359 | Inf | up |
| Methanosarcina acetivorans | 0.006359 | 0.013853 | down |
| Neisseria cinerea | 0.006967 | 25.71258 | up |
| Streptomyces sp. SPB074 | 0.007018 | 0.014033 | down |
| Debaryomyces fabryi | 0.009669 | 0.259087 | down |
| Bovine gammaherpesvirus 4 | 0.011323 | 0.11742 | down |
| Actinomyces viscosus | 0.011675 | Inf | up |
| Actinomyces sp. oral taxon 175 | 0.011675 | Inf | up |
| Actinomyces sp. oral taxon 172 | 0.011675 | Inf | up |
| Porphyromonas uenonis | 0.011675 | Inf | up |
| Neisseria weaveri | 0.011675 | Inf | up |
| Kingella kingae | 0.011675 | Inf | up |
| Capnocytophaga sp. oral taxon 412 | 0.011675 | Inf | up |
| Streptococcus cristatus | 0.013462 | 63.9133 | up |
| Schizopora paradoxa | 0.013644 | 0.174177 | down |
| Paenibacillus jilunlii | 0.013861 | 0.550113 | down |
| Veillonella tobetsuensis | 0.014386 | 687.1982 | up |
| Porphyromonas sp. oral taxon 279 | 0.015459 | 13.10104 | up |
| Fomitiporia mediterranea | 0.015598 | 0.034704 | down |
| Bacillus kribbensis | 0.015611 | 2.462143 | up |
| Penicillium expansum | 0.01633 | 2.225929 | up |
| Aggregatibacter actinomycetemcomitans | 0.016961 | 32.11072 | up |
| Aggregatibacter segnis | 0.018132 | 9.643777 | up |
| Succinispira mobilis | 0.020269 | 406.8413 | up |
| Streptomyces virginiae | 0.020269 | 0.105339 | down |
| Veillonella parvula | 0.020603 | 24.27128 | up |
| Pasteurella multocida | 0.021094 | Inf | up |
| Veillonella sp. 6_1_27 | 0.021094 | Inf | up |
| Pasteurella bettyae | 0.021094 | Inf | up |
| Atopobium vaginae | 0.021825 | 0.198116 | down |
| Enterococcus faecalis | 0.022011 | 98.29258 | up |
| Veillonella dispar | 0.022192 | 30.80763 | up |
| Porphyromonas somerae | 0.0236 | 11.34495 | up |
| Pseudomonas aeruginosa | 0.023971 | 1.280849 | up |
| Neisseria flavescens | 0.02425 | 12.98724 | up |
| Prevotella fusca | 0.024581 | 6.773298 | up |
| Haemophilus influenzae | 0.025788 | 22.24756 | up |
| Rickettsia prowazekii | 0.02589 | 0.200439 | down |
| Neisseria perflava | 0.02589 | 16.28304 | up |
| Campylobacter jejuni | 0.02748 | 1.455775 | up |
| Porphyromonas macacae | 0.028429 | 102.3076 | up |
| Rodentibacter pneumotropicus | 0.02858 | 38.58184 | up |
| Prevotella buccae | 0.029044 | 9.711888 | up |
| Halomonas huangheensis | 0.029231 | 2.044871 | up |
| Prevotella salivae | 0.030286 | 7.442735 | up |
| Bacteroides ovatus | 0.030286 | 8.715686 | up |
| Equid gammaherpesvirus 2 | 0.030346 | 0.234086 | down |
| Neisseria meningitidis | 0.03083 | 12.2346 | up |
| Streptococcus infantis | 0.030952 | 36.14919 | up |
| Aggregatibacter sp. oral taxon 458 | 0.032004 | 26.65447 | up |
| Botrytis cinerea | 0.032812 | 0.251692 | down |
| Haemophilus parainfluenzae | 0.032815 | 29.2425 | up |
| Prevotella falsenii | 0.033174 | 9.574487 | up |
| Neisseria lactamica | 0.03324 | 12.15806 | up |
| Spizellomyces punctatus | 0.033984 | 7.168129 | up |
| Prevotella sp. C561 | 0.034358 | 21.00752 | up |
| Vibrio vulnificus | 0.036076 | 0.478273 | down |
| Prevotella sp. ICM33 | 0.036183 | 15.49814 | up |
| Stereum hirsutum | 0.037079 | 0.01328 | down |
| Penicillium italicum | 0.037079 | 0.087571 | down |
| Actinomadura oligospora | 0.037079 | 0.022906 | down |
| Red seabream iridovirus | 0.037079 | 0.024071 | down |
| Cutaneotrichosporon oleaginosus | 0.037079 | 0.034636 | down |
| Kirsten murine sarcoma virus | 0.037079 | 0.044522 | down |
| Actinomyces sp. ICM39 | 0.037629 | Inf | up |
| Rothia aeria | 0.037629 | Inf | up |
| [Clostridium] symbiosum | 0.037629 | Inf | up |
| Gemella sanguinis | 0.037629 | Inf | up |
| Capnocytophaga sp. oral taxon 380 | 0.037629 | Inf | up |
| Fusobacterium canifelinum | 0.037629 | Inf | up |
| Haemophilus haemolyticus | 0.037813 | 25.2384 | up |
| Veillonella atypica | 0.037869 | 26.59604 | up |
| Haemophilus parahaemolyticus | 0.037896 | 10.26039 | up |
| Prevotella loescheii | 0.037979 | 48.66516 | up |
| Haemophilus sputorum | 0.037979 | 673.742 | up |
| Gallibacterium anatis | 0.037979 | 263.2893 | up |
| Neisseria shayeganii | 0.037979 | 595.3159 | up |
| Neisseria sp. oral taxon 014 | 0.038546 | 39.52916 | up |
| Candida albicans | 0.038875 | 1.859191 | up |
| Bacillus thuringiensis | 0.039722 | 0.624929 | down |
| Paenibacillus odorifer | 0.040188 | 0.586087 | down |
| Prevotella scopos | 0.04045 | 15.03176 | up |
| [Candida] auris | 0.040561 | 0.123785 | down |
| Trichoderma reesei | 0.040561 | 0.021765 | down |
| Isosphaera pallida | 0.040561 | 0.094865 | down |
| Chaetomium thermophilum | 0.040561 | 0.078903 | down |
| Pleurotus ostreatus | 0.040561 | 0.070517 | down |
| Aspergillus nidulans | 0.040561 | 0.055323 | down |
| Alphapapillomavirus 7 | 0.040561 | 0.089899 | down |
| Malassezia pachydermatis | 0.040561 | 0.052858 | down |
| Rhizophlyctis rosea | 0.040561 | 0.050814 | down |
| uncultured prokaryote | 0.041039 | 10.32233 | up |
| Porphyromonas gingivicanis | 0.041111 | 76.95276 | up |
| Prevotella oralis | 0.041111 | 53.54028 | up |
| Avian leukosis virus | 0.04126 | 0.827546 | down |
| Waddlia chondrophila | 0.042386 | 0.600474 | down |
| Candidatus Pelagibacter ubique | 0.044316 | 0.069773 | down |
| Nocardia jiangxiensis | 0.044316 | 0.017353 | down |
| Pneumocystis jirovecii | 0.044316 | 0.121598 | down |
| Thermosynechococcus elongatus | 0.044316 | 0.075935 | down |
| Porphyromonas endodontalis | 0.044419 | 6.027089 | up |
| Prevotella denticola | 0.046435 | 32.70058 | up |
| Capnocytophaga sp. oral taxon 332 | 0.046648 | 13.3029 | up |
| Mycobacterium tuberculosis | 0.047081 | 0.777962 | down |
| Rhizophagus irregularis | 0.047555 | 0.824146 | down |
| Erythrobacter litoralis | 0.048361 | 0.051498 | down |
| Aspergillus flavus | 0.048361 | 0.040464 | down |
| Exophiala mesophila | 0.048361 | 0.186959 | down |
| [Enterobacter] aerogenes | 0.048721 | 2.080883 | up |
| Neisseria sp. GT4A_CT1 | 0.049906 | 6.399039 | up |
